# Salusin-*β* Is Involved in Diabetes Mellitus-Induced Endothelial Dysfunction via Degradation of Peroxisome Proliferator-Activated Receptor Gamma

**DOI:** 10.1155/2017/6905217

**Published:** 2017-11-19

**Authors:** Hai-Jian Sun, Dan Chen, Pei-Yao Wang, Ming-Yu Wan, Chen-Xing Zhang, Zhi-Xuan Zhang, Wei Lin, Feng Zhang

**Affiliations:** ^1^Department of Physiology, Nanjing Medical University, Nanjing, Jiangsu 211166, China; ^2^Department of Basic Medicine, Wuxi School of Medicine, Jiangnan University, Wuxi, Jiangsu 214122, China

## Abstract

The pathophysiological mechanisms for vascular lesions in diabetes mellitus (DM) are complex, among which endothelial dysfunction plays a vital role. Therapeutic target against endothelial injury may provide critical venues for treatment of diabetic vascular diseases. We recently identified that salusin-*β* contributed to high glucose-induced endothelial cell apoptosis. However, the roles of salusin-*β* in DM-induced endothelial dysfunction remain largely elusive. Male C57BL/6J mice were used to induce type 2 diabetes mellitus (T2DM) model. Human umbilical vein endothelial cells (HUVECs) were cultured in high glucose/high fat (HG/HF) medium. We demonstrated increased expression of salusin-*β* in diabetic aortic tissues and high-glucose/high-fat- (HG/HF-) incubated HUVECs. Disruption of salusin-*β* by shRNA abrogated the reactive oxygen species (ROS) production, inflammation, and nitrotyrosine content of HUVECs cultured in HG/HF medium. The HG/HF-mediated decrease in peroxisome proliferator-activated receptor *γ* (PPAR*γ*) expression was restored by salusin-*β* shRNA, and PPAR*γ* inhibitor T0070907 abolished the protective actions of salusin-*β* shRNA on endothelial injury in HG/HF-treated HUVECs. Salusin-*β* silencing obviously improved endothelium-dependent vasorelaxation, oxidative stress, inflammatory response, and nitrative stress in diabetic aorta. Taken together, our results highlighted the essential role of salusin-*β* in pathological endothelial dysfunction, and salusin-*β* may be a promising target in treatment of vascular complications of DM.

## 1. Introduction

Type 2 diabetes mellitus (T2DM) is a tremendous threat to human health around the world [1, 2]. Patients with DM had higher risk for cardiovascular events and complications than nondiabetic patients [3]. The impaired function in endothelium is critically involved in many cardiovascular disorders such as atherosclerosis, hypertension, and DM [4]. Endothelial dysfunction is a core event in the development of DM and determines future vascular diseases' complications [5]. Improvement of endothelial dysfunction may be an attractive therapeutic strategy for treatment of DM-related cardiovascular disorders [4]. Decreased nitric oxide (NO) bioavailability is one of the earliest pathologic events that contributes to the development and progression of diabetic vascular complications [6]. It is well accepted that NADPH oxidase-derived reactive oxygen species (ROS) are requisite mediators of vascular injury in diabetes [7, 8]. Nitrative stress (excessive production of NO and peroxynitrite) is presented in diabetic aorta, which leads to endothelial dysfunction [9–11]. Chronic inflammation in endothelial cells is associated with endothelial function disruption in DM [12, 13].

Salusin-*β* is an endogenous bioactive peptide with 20 amino acid residues [14]. Salusin-*β* stimulates the proliferation, migration of vascular smooth muscle cells (VSMCs), vascular fibrosis, and foam cell formation of VSMCs [15–17]. Salusin-*β* acts as a potential proatherogenic factor via promoting macrophage foam cell formation [18]. Central microinjection of salusin-*β* contributes to sympathetic activation, norepinephrine release, and hypertension [19–22]. The positive stains for salusin-*β* have been detected in vasculature including endothelium [23]. Salusin-*β* blockade ameliorates endothelial inflammation to improve pulmonary arterial hypertension and pulmonary vascular remodeling [24]. Plasma salusin-*β* levels are higher in subjects with DM than healthy controls [25]. A recent study reveals that inhibition of salusin-*β* alleviates oxidative stress, inflammation, and cardiac dysfunction in diabetic rats [26]. Furthermore, we recently identified that salusin-*β* contributed to high glucose-induced inhibition of proliferation, migration, and angiogenesis in endothelial cells [27]. Up to now, the molecular link between salusin-*β* and DM-related endothelial injury has yet to be fully clarified. Therefore, we investigated the potential roles of salusin-*β* in endothelial dysfunction in DM and the underlying molecular mechanisms.

## 2. Material and Methods

### 2.1. Animals and Experimental Groups

Male C57BL/6J mice aged 6 weeks (Vital River Biological, Beijing, China) were used to induce type 2 diabetic model. All experiments were conformed to the rules and regulations of the Experimental Animal Care and Use Committee of Jiangnan University. All experiments adhered to the Care and Use of Laboratory Animal published by the US National Institutes of Health (NIH publication, 8th edition, 2011). The mice were housed on 12 : 12 hour light-dark cycle in a temperature-controlled and humidity-controlled room, with free access to standard chow and tap water. The animal model for type 2 diabetes in mice was made as previously described [28–30]. In brief, a combination of low-dose streptozotocin (STZ) and a high-fat diet (HFD) was applied to make an ideal animal model for type 2 diabetes in mice. After 2 weeks of acclimatization, all mice were randomly divided into four groups (*n* = 8 for each group). Two groups of mice received an intraperitoneal injection of vehicle and were fed with a normal diet (14.7 kJ/g, 13% of energy as fat) for 10 weeks. Another two groups of mice were subject to 4 h fasting and then were subjected to intraperitoneal injection of low-dose streptozotocin (STZ, 120 mg/kg body weight in 10 mmol/l citrate buffer, pH 4.0); the normal diet was replaced with high-fat diet (21.8 kJ/g, 60% of energy as fat; D12492, Research Diets, New Brunswick, NJ, USA) for 7 weeks three weeks after injection of STZ. Eight weeks after the injection of STZ, the mice in control and diabetic mice received intravenous injection of adenoviral vectors encoding scramble shRNA (Ad-Scr-shRNA, 2.0 × 10^10^ plaque-forming units) or adenoviral vectors encoding salusin-*β* shRNA (Ad-Salusin-*β* shRNA, 2.0 × 10^10^ plaque-forming units). The Ad-Scr shRNA and Ad-Salusin-*β* shRNA were constructed and purchased from Genomeditech Co. (Shanghai, China). Two weeks after introduction of Ad-Scr shRNA and Ad-Salusin-*β* shRNA, all mice were euthanized with an overdose of pentobarbital sodium (150 mg/kg, i.v.). The body weight or fasting blood glucose was measured at the end of experiments before sacrifice. The measurement for insulin tolerance test (ITT) and glucose tolerance test (GTT) in each mouse was conducted as in our previous reports [26, 28, 30].

### 2.2. Evaluation of Vasorelaxation

Endothelial function was assessed by the vasorelaxation response to acetylcholine (ACh), an endothelium-dependent vasodilator, with that of sodium nitroprusside (SNP), an endothelium-independent vasodilator, respectively. In brief, the aorta from mice was collected in ice-cold physiological saline solution (PSS) and cut into 4 rings of 1 mm in length. The aortic rings were mounted onto two stainless steel wires and installed on a wire myograph (Model 620M, Danish Myo Technology, Aarhus, Denmark), filled with 5 ml PSS buffer aerated with 95% O_2_ and 5% CO_2_ at 37°C, which linked to transducers to recorded data. The artery segments were equilibrated for 60 min with an optimal initial tension before the experiments were started. After equilibration, aortic rings were contracted by phenylephrine (PE, 1 *μ*M) to obtain similar precontraction among different groups. Once a stable contraction was achieved, the rings were exposed to cumulative concentrations of ACh (10^−9^ to 10^−4^ M). The aortic rings were then washed and equilibrated to baseline levels, and the procedure was repeated with SNP of 10^−9^ to 10^−5^ M. Relaxation at each concentration was presented as the percentage of force generated in response to PE [31–33].

### 2.3. Cell Culture

Human umbilical vein endothelial cells (HUVECs) were cultured in RPMI 1640 medium supplemented with 10% fetal bovine serum, 100 units/ml penicillin, and 100 *μ*g/ml streptomycin under a condition at 37°C in a humidified air containing 5% CO_2_. In order to mimic the increased glucose level in diabetes, HUVECs were cultured in high glucose/high fat (HG/HF) medium, containing glucose (25 mM) and saturated free fatty acid (FFA) palmitate (16:C; 500 *μ*M, Sigma, USA) for 24 h according to the previous reports [9, 34]. For *in vitro* studies, HUVECs were subcultured in six-well plates and transfected with Ad-Scr shRNA and Ad-Salusin-*β* shRNA (1 × 10^8^ plaque-forming units/ml) for 24 h as previously described [35].

### 2.4. Immunofluorescence Staining

After fixed with 4% paraformaldehyde for 30 min, HUVECs were permeabilized with 0.1% Triton X-100 in PBS for 15 min. Cells were blocked with 5% bovine serum albumin (BSA) for 1 h at room temperature and then incubated with indicated anti-nitrotyrosine antibody at 4°C overnight. After three washes with PBS, cells were detected with goat anti-rabbit IgG H&L Alexa Fluor® 488. Finally, nuclei were stained with 4′,6-diamidino-2-phenylindole (DAPI) for 10 min. Images were acquired by a fluorescence microscope (80i, Nikon, Tokyo, Japan).

### 2.5. Immunohistochemistry Staining

The thoracic aorta was rinsed with cool sterile phosphate buffered saline (PBS). Paraffin-embedded heart and blood vessel sections (5 *μ*m) were permeabilized using 0.1% Triton X-100 for 10 min after deparaffinization and rehydration. The sections were washed in PBS then blocked with 10% goat serum for 1 h, then incubated with rabbit anti-salusin-*β* antibody overnight at 4°C, and were then visualized under a Nikon microscope digital camera system (Nikon, Tokyo, Japan).

### 2.6. Enzyme-Linked Immunosorbent (ELISA) Assay

ELISA assays were performed to measure salusin-*β* level in stimulated HUVECs according to the manufacturer's protocols (USCN Life Science, Houston, TX, USA). The protein levels of TNF-*α*, IL-1*β*, VCAM-1, and MCP-1 in medium supernatant from each sample were detected by commercial ELISA kits (BOSTER, Wuhan, China) according to the manufacturer's instructions as previously described [36, 37]. The blanks, diluted standards, or samples were added appropriately into coat wells in 96-well plates and HRP-conjugated antibody was coincubated at 37°C for 30 min. The reaction system was terminated with stopped solution, and the absorbance was determined using a microplate reader (STNERGY/H4, BioTek, Vermont, USA).

### 2.7. Real-Time PCR

Total RNA was extracted using TRIzol reagent according to the manufacturer's instructions. The equal RNA was used to generate cDNA using a first-strand cDNA Synthesis Kit (CWBIO, Taizhou, Jiangsu, China). The real-time quantitative PCR was performed in triplicates by using SYBR® mixture (CWBIO, Taizhou, Jiangsu, China). The average cycle thresholds (Ct) were employed to quantify fold change. The 2^−△△CT^ method was reported to calculate relative gene expression levels. The primer sequences used for real-time PCR were listed in the supplemental table (Tables S1 and S2 available online at https://doi.org/10.1155/2017/6905217).

### 2.8. Western Blot

The protein in collected HUVECs or aorta was extracted in RIPA lysis, and equal amounts of total proteins electrophoresed, blotted, and then incubated with required primary antibodies including endothelial nitric oxide synthase (eNOS), phosphorylated-eNOS (p-eNOS), inducible nitric oxide synthase (iNOS), nitrotyrosine, NOX-2, p22^phox^, p47^phox^, peroxisome proliferator-activated receptor *γ* (PPAR*γ*), glyceraldehyde phosphate dehydrogenase (GAPDH), interleukin-1*β* (IL-1*β*), monocyte chemoattractant protein 1 (MCP-1), tumor necrosis factor-*α* (TNF-*α*), and vascular cellular adhesion molecule-1 (VCAM-1) at 4°C overnight. The blots were then incubated with appropriate secondary horseradish peroxidase- (HRP-) conjugated antibodies, and the immunoreactive proteins were visualized by enhanced chemiluminescence (Millipore Darmstadt, Germany).

### 2.9. Intracellular ROS Measurement

The collected HUVECs or aorta sections were fixed and treated with dihydroethidium (DHE, 10 *μ*M) or 2′,7′-dichlorofluorescin diacetate (DCFH-DA, 10 *μ*M) for 20 min at 37°C, respectively. The fluorescence signals were captured with a multidetection microplate reader and quantified with the Image-Pro Plus 6.0 (version 6.0, Media Cybernetics, Bethesda, MD, USA) by using the same parameters [16].

### 2.10. Total NO Production and Nitrotyrosine Content Measurement

The total basal NOx (NO and its oxidative metabolic products, NO_2_^−^ and NO_3_^−^) production was detected by a NO detection kit (Beyotime Biotech Inc., Nanjing, China) was used following the instructions by the manufacturer. The total aortic proteins were measured using a BCA protein assay kit (Beyotime Institute of Biotechnology, China). NO data was expressed in nM/mg protein [9, 31]. The determination of nitrotyrosine content, which is taken as an index of protein nitration and nitrative stress, was performed with the aid of a Nitrotyrosine Assay Kit (Millipore, MA, USA) according to the manufacturer's instruction. Nitrotyrosine level was presented as pM/mg protein [6, 9].

### 2.11. Assay of eNOS Activity

The activity of eNOS in collected HUVECs was assessed by the conversion of L-arginine to NO using a Nitric Oxide Synthase Assay Kit (Beyotime Biotech Inc., Nanjing, China) as previously described [38, 39].

### 2.12. Chemicals

HUVECs were purchased from American Type Culture Collection (Rockville, MD, USA). Nitric Oxide Synthase Assay Kit, DHE, and DCFH-DA were obtained from Beyotime Institute of Biotechnology (Shanghai, China). Antibodies against eNOS, p-eNOS, and iNOS were obtained from Cell Signaling Technology (Beverly, MA, USA). Antibodies against nitrotyrosine, NOX-2, p22^phox^, p47^phox^, and goat anti-rabbit IgG H&L (Alexa Fluor 488) were purchased from Abcam (Cambridge, MA, USA). Antibody against salusin-*β* was obtained from Bachem (Bubendorf, Switzerland). Antibodies against PPAR*γ*, GAPDH, IL-1*β*, MCP-1, TNF-*α*, and VCAM-1 and HRP-conjugated secondary antibodies were purchased from Proteintech Group Inc. (Wuhan, China). 2-Chloro-5-nitro-N-4-pyridinyl-benzamide (T0070907) was purchased from Cayman Chemical Co. (Ann Arbor, MI, USA). The specific primers were synthesized by Sangon Biotech Co. Ltd. (Shanghai, China).

### 2.13. Statistical Analysis

All results were defined as mean ± SE. Comparisons within two groups were made by Student's *t*-test. Statistical analysis was performed by ANOVA/Dunnet *t*-test for multiple group comparisons. The criterion for statistical significance was set at *P* < 0.05.

## 3. Results

### 3.1. HG/HF Induced Salusin-*β* Expression in HUVECs

Immunofluorescence staining showed the upregulated salusin-*β* expression in HUVECs exposed to HG/HF for 24 h (Figure S1(a)). HG/HF treatment not only enhanced salusin-*β* protein expression (Figure S1(b)) but also elevated salusin-*β* mRNA level (Figure S1(c)) in HUVECs, as detected by ELISA or real-time PCR, respectively. These results revealed that HG/HF is an inducer of salusin-*β* expression in HUVECs.

### 3.2. Salusin-*β* Blockade Rectified Oxidative Stress in HUVECs

**Figure 1:**
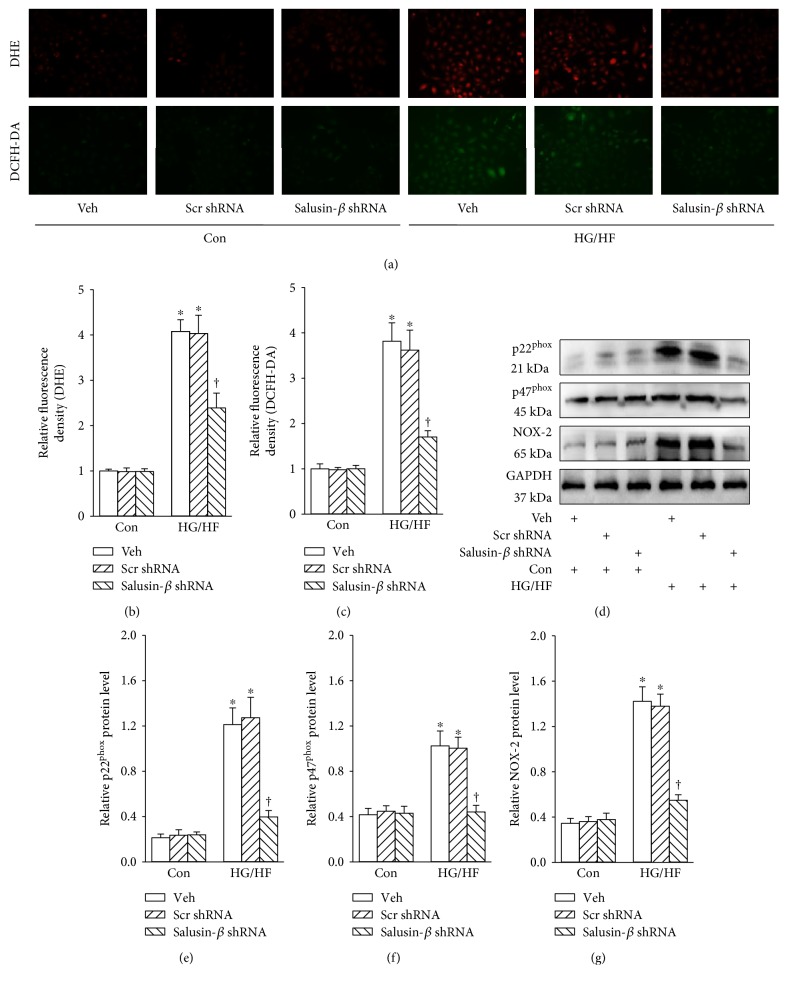
Effects of salusin-*β* knockdown on the oxidative stress of HUVECs. (a, c) Images showing the levels of superoxide anions detected by DHE staining. (b, d) Images showing the ROS levels detected by DCFH-DA staining. (d) Blots showing the protein expressions of p22^phox^, p47^phox^, and NOX-2. Bar graph showing the relative quantification of p22^phox^ (e), p47^phox^ (f), and NOX-2 (g). Values are mean ± SE. ^∗^*P* < 0.05 versus control (Con), ^†^*P* < 0.05 versus vehicle (Veh) or scramble (Scr) shRNA. *n* = 6 for each group.

HG/HF caused the overproduction of ROS in HUVECs as evidenced by DHE (Figures 1(a) and 1(b)) or DCFH-DA (Figures 1(a) and 1(c)) fluorescent dye, which was abrogated by inhibition of salusin-*β*. The upregulated protein expressions of NADPH oxidase subunits p22^phox^ (Figures 1(d) and 1(e)), p47^phox^ (Figures 1(d) and 1(f)), and NOX-2 (Figures 1(d) and 1(g)) were observed in HUVECs response to HG/HF, and these changes were prevented by salusin-*β* knockdown.

### 3.3. Salusin-*β* Blockade Retarded Nitrative Stress *In Vitro* and Improved Endothelial-Dependent Vasorelaxation *In Vivo*

**Figure 2:**
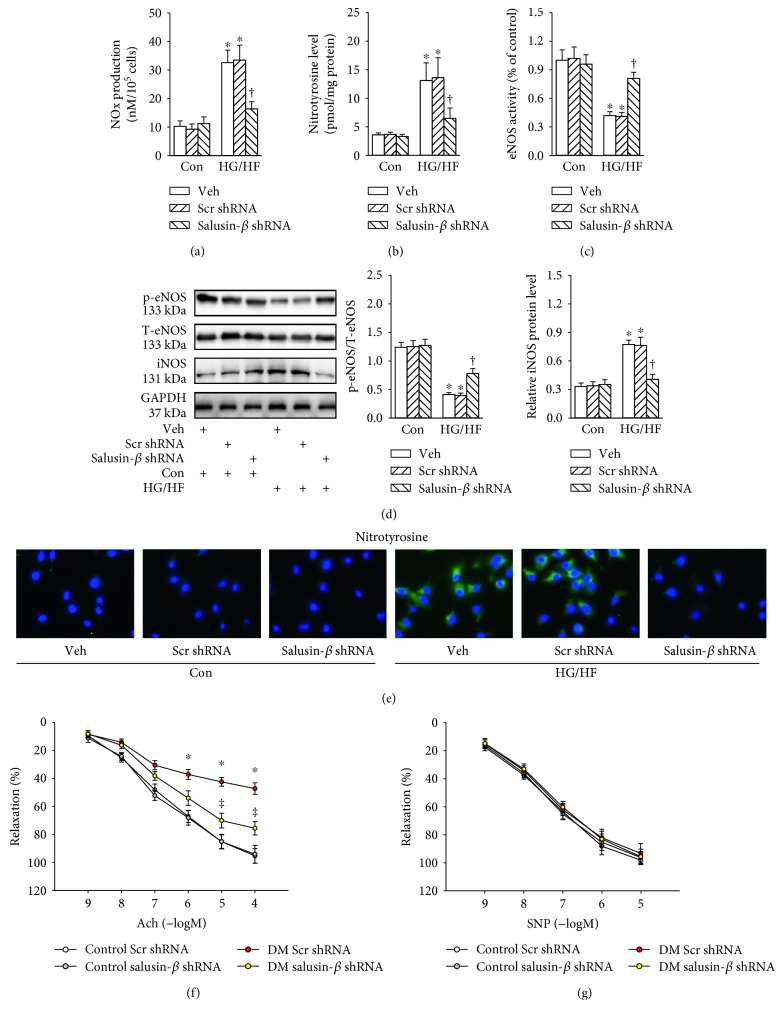
Effects of salusin-*β* knockdown on the nitrative stress of HUVECs. (a) Total NOx levels. (b) Nitrotyrosine formation. (c) eNOS activity. (d) Blots showing the protein expressions of phosphorylated eNOS and iNOS. (e) The nitrotyrosine formation based on immunofluorescent staining. Salusin-*β* silencing improved the impaired endothelium-dependent vasorelaxation in type 2 diabetic mice (f), but had minimal effects on endothelium-independent vasorelaxation (g). Values are mean ± SE. ^∗^*P* < 0.05 versus control (Con) or control scramble (Scr) shRNA, ^†^*P* < 0.05 versus vehicle (Veh), scramble (Scr) shRNA or DM scramble (Scr) shRNA. *n* = 6 for each group.

Incubation of HUVECs with HG/HF promoted NOx production (Figure 2(a)), nitrotyrosine content (Figure 2(b)), but decreased the eNOS activity (Figure 2(c)), and these changes were reversed by partial deletion of salusin-*β*. The phosphorylated eNOS level was obviously downregulation, while iNOS expression was markedly increased in HG/HF-treated HUVECs compared with those from vehicle-treated cells (Figure 2(d)). Immunofluorescence to nitrotyrosine-positive cells further demonstrated that nitrotyrosine was significantly increased after HG/HF treatment, which was inhibited by salusin-*β* shRNA (Figure 2(e)). Moreover, in comparison with the aortic segments from control mice, the diabetic aortic segments exhibited impaired Ach-induced vasorelaxation. Interestingly, the aorta from diabetic mice with salusin-*β* silencing exhibited improved ACh-induced vasorelaxation compared with vehicle (Figure 2(f)). The endothelium-independent vasodilator responses to SNP were not different among groups (Figure 2(g)). These data indicated that knockdown of salusin-*β* exerted direct endothelial protective effects.

### 3.4. Salusin-*β* Blockade Attenuated Inflammation in HG/HF-Treated HUVECs

**Figure 3:**
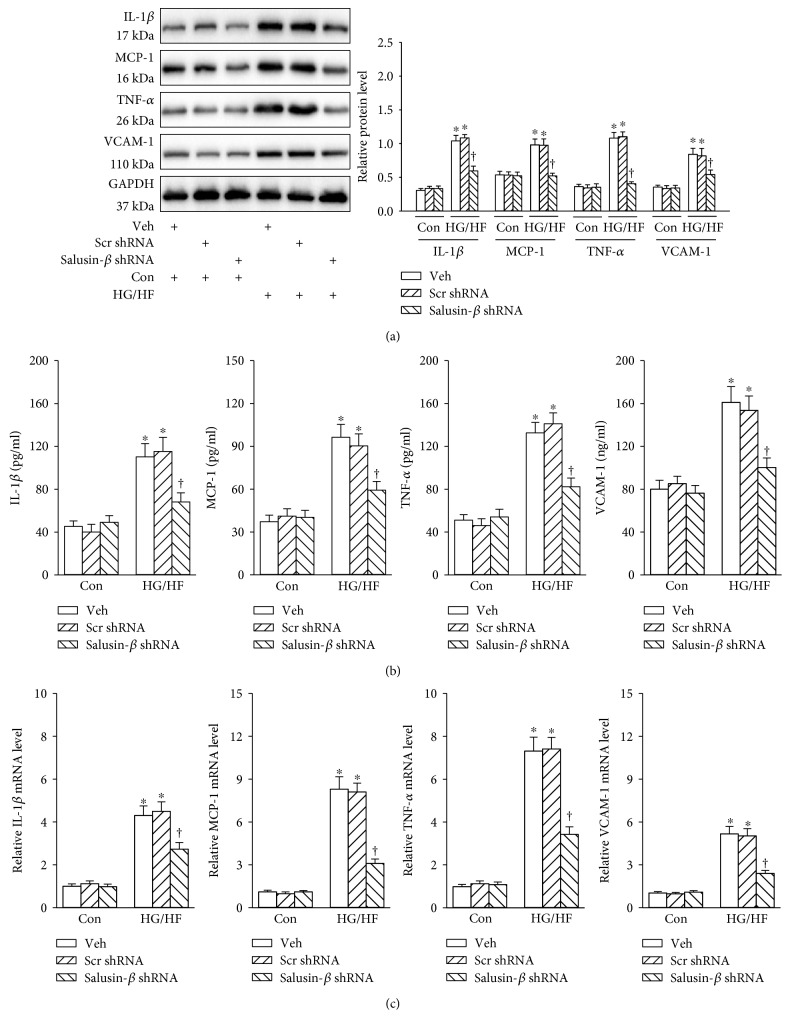
Effects of salusin-*β* knockdown on the inflammation of HUVECs. (a) Blots showing the protein expressions of IL-1*β*, MCP-1, TNF-*α*, and VCAM-1. (b) The protein levels of IL-1*β*, MCP-1, TNF-*α*, and VCAM-1 determined with ELISA. (c) The mRNA levels of IL-1*β*, MCP-1, TNF-*α*, and VCAM-1 determined with real-time PCR. Values are mean ± SE. ^∗^*P* < 0.05 versus control (Con), ^†^*P* < 0.05 versus vehicle (Veh) or scramble (Scr) shRNA. *n* = 6 for each group.

The protein expressions of inflammatory molecules including IL-1*β*, MCP-1, TNF-*α*, and VCAM-1 were increased in HUVECs response to HG/HF, and silencing of salusin-*β* with shRNA eliminated HG/HF-mediated inflammatory response in HUVECs (Figure 3(a)). The similar results were also observed by ELISA (Figure 3(b)) or real-time PCR assay (Figure 3(c)).

### 3.5. PPAR*γ* Participated in the Effects of Salusin-*β* Blockade on Nitrative Stress in HUVECs

**Figure 4:**
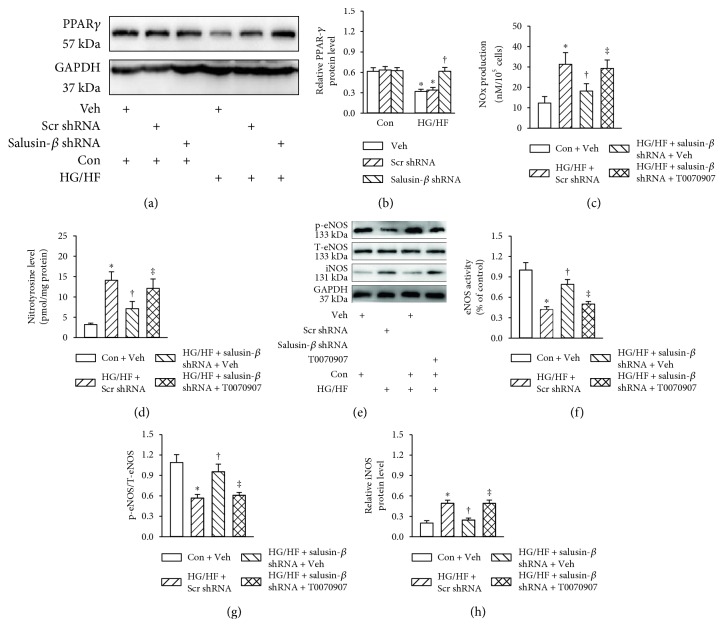
PPAR*γ* participated in the effects of salusin-*β* blockade on nitrative stress in HG/HF-treated HUVECs. HUVECs were pretreated with T0070907 (10 *μ*M) for 6 h, and transfected with adenoviral vectors encoding salusin-*β* shRNA or control shRNA for 24 h, and then cultured in control or HG/HF-containing medium for 24 h. (a) Blots showing the protein expressions of PPAR*γ*. (b) Total NOx levels. (c) nitrotyrosine formation. (d) Blots showing the protein expressions of phosphorylated eNOS and iNOS. (e) eNOS activity. Bar group showing the relative quantification of phosphorylated eNOS (f) and iNOS (g). Values are mean ± SE. ^∗^*P* < 0.05 versus control (Con) + vehicle (Veh), ^†^*P* < 0.05 versus HG/HF + scramble (Scr) shRNA, ^‡^*P* < 0.05 versus HG/HF + salusin-*β* shRNA + vehicle (Veh). *n* = 6 for each group.

The protein level of PPAR*γ* was higher in vehicle-treated cells, but it was obviously decreased in HG/HF-incubated HUVECs, and shRNA-mediated silencing of salusin-*β* restored the PPAR*γ* protein expression in HUVECs stimulated by HG/HF (Figures 4(a) and 4(b)). These results unveiled that salusin-*β* may act on PPAR*γ* to affect HG/HF-induced endothelial injury. Therefore, we investigated the effect of PPAR*γ* inhibitor T0070907 on endothelial injury in HG/HF-exposed HUVECs. Chronic treatment with T0070907 abolished the protective effects of salusin-*β* blockade on NOx production (Figure 4(c)), nitrotyrosine content (Figure 4(d)), eNOS activity (Figure 4(f)), phosphorylated eNOS level (Figures 4(e) and 4(g)), and iNOS expression (Figures 4(e) and 4(h)) in HUVECs incubated by HG/HF.

### 3.6. PPAR*γ* Participated in the Effects of Salusin-*β* Blockade on Oxidative Stress and Inflammation in HUVECs

Preincubation of HUVECs with T0070907 counteracted the effects of salusin-*β* silencing on protein expressions of NADPH oxidase subunits p47^phox^ (Figure S2(a) and S2(b)), p22^phox^** (**Figure S2(a) and S2(c)), NOX-2 (Figure S2(a) and S2(d)), and inflammatory molecules including IL-1*β*, MCP-1, TNF-*α*, and VCAM-1(Figure S2(a) and S2(e)) in HUVECs upon HG/HF stimulation. ELISA (Figure S2(f)) or real-time PCR assay (Figure S2(g)) furthermore demonstrated that the protective actions of salusin-*β* blockade on HG/HF-triggered inflammation were diminished by PPAR*γ* inhibitor T0070907. When it came to oxidative stress, DHE (Figure S3(a) and S3(c)) or DCFH-DA (Figure S3(b) and S3(d)) fluorescent staining showed that PPAR*γ* inhibitor T0070907 impeded the beneficial effect of salusin-*β* blockade on HG/HF-evoked superoxide anions overproduction in HUVECs.

### 3.7. Salusin-*β* Expression in Aorta of Diabetic Mice In Vivo

**Figure 5:**
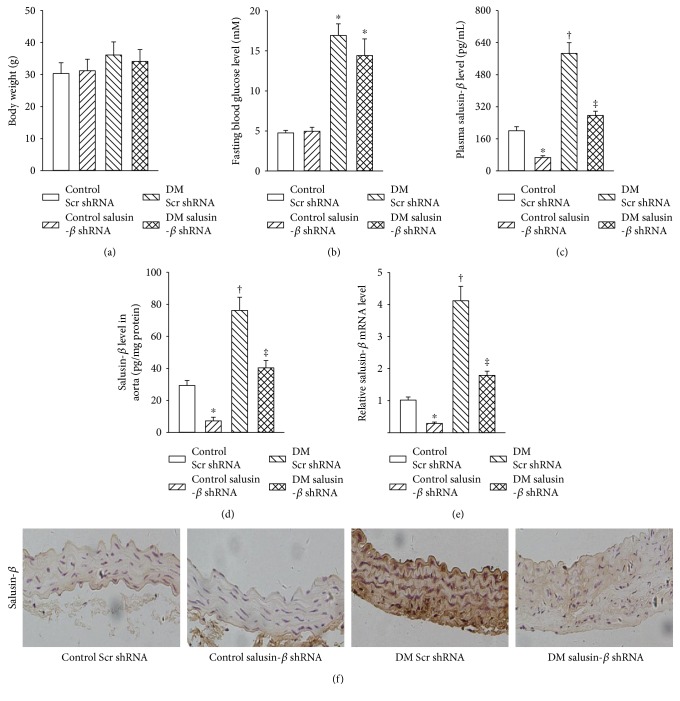
Salusin-*β* expression in aorta of diabetic mice *in vivo*. Salusin-*β* silencing had no effect the bodyweight (a) and fasting blood glucose (b) in type 2 diabetic mice. (c) Plasma salusin-*β* level. (d) Salusin-*β* level in aorta from mice determined with ELISA. (e) Salusin-*β* level in aorta from mice determined with real-time PCR. (f) Salusin-*β* in aorta from mice detected by immunohistochemistry. Values are mean ± SE. ^∗^*P* < 0.05 versus control scramble (Scr) shRNA, ^†^*P* < 0.05 versus control scramble (Scr) shRNA or control salusin-*β* shRNA, ^‡^*P* < 0.05 versus DM scramble (Scr) shRNA. *n* = 7 for each group.

*In vitro* results demonstrated that silencing of salusin-*β* attenuated endothelial cell damage in response to HG/HF. Therefore, mice model of T2DM was made to investigate whether the expression of salusin-*β* was altered in aorta of diabetic mice. There was no significant difference in body weight (Figure 5(a)) or fasting blood glucose (Figure 5(b)) in both control and diabetic mice. The impaired insulin tolerance test (ITT) and glucose tolerance test (GTT) in diabetic mice were not affected by intravenous injection of adenoviral vectors encoding salusin-*β* shRNA (Figure S4), implying that salusin-*β* may not be involved in the destructive glucose excursion and insulin sensitivity in diabetic mice. The plasma salusin-*β* (Figure 5(c)), protein (Figure 5(d)), and mRNA (Figure 5(e)) levels of salusin-*β* in aorta were higher in diabetic mice than in control mice, which were obviously reduced by salusin-*β* shRNA in both diabetic mice and control mice. Immunohistochemistry results further established that the upregulated salusin-*β* level in aorta of diabetic mice was decreased by knockdown of salusin-*β* (Figure 5(f)).

### 3.8. Salusin-*β* Blockade Attenuated Nitrative Stress and Inflammation *In Vivo*

**Figure 6:**
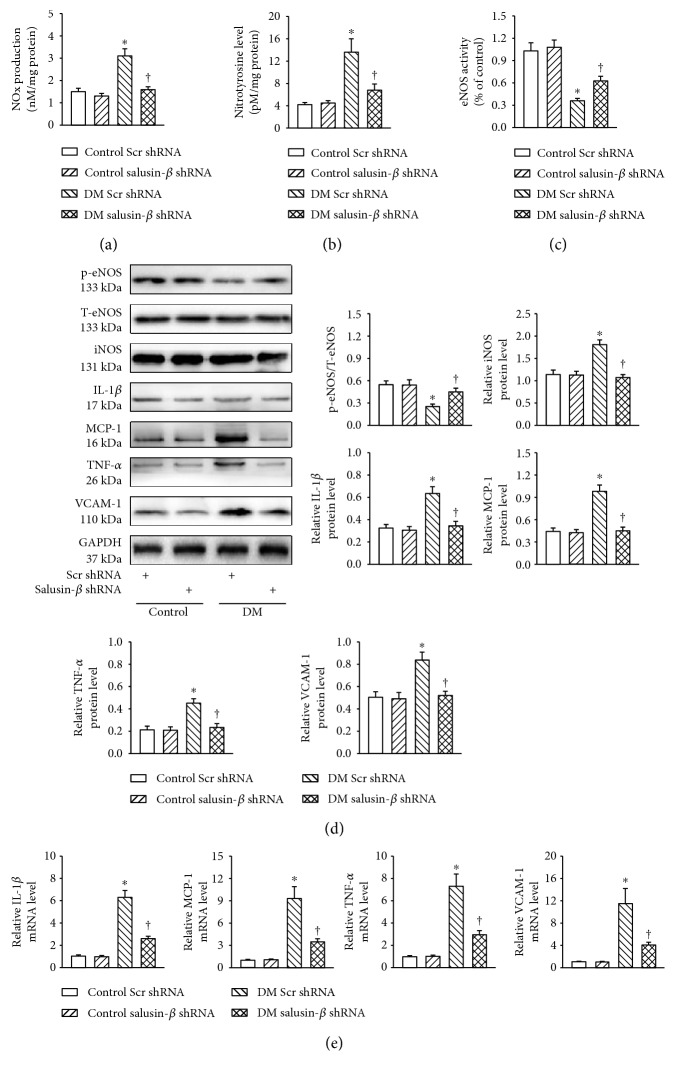
Salusin-*β* blockade attenuated nitrative stress and inflammation *in vivo*. Intravenous injection of adenoviral vectors encoding salusin-*β* shRNA (Ad-Salusin-shRNA, 2.0 × 10^10^ plaque-forming units) or scramble shRNA (Ad-Scr-shRNA) were carried out 8 weeks after STZ injection. The measurements were made 2 weeks after the first adenovirus transfer. (a) Total NOx levels. (b) nitrotyrosine formation. (c) eNOS activity. (d) Represented blots showing the protein expressions of phosphorylated eNOS, iNOS, IL-1*β*, MCP-1, TNF-*α*, and VCAM-1. (e) Bar group showing the mRNA levels of IL-1*β*, MCP-1, TNF-*α*, and VCAM-1 determined with real-time PCR. Values are mean ± SE. ^∗^*P* < 0.05 versus control scramble (Scr) shRNA or control salusin-*β* shRNA, ^†^*P* < 0.05 versus DM scramble (Scr) shRNA. *n* = 7 for each group.

The production of NOx (Figure 6(a)) and nitrotyrosine level (Figure 6(b)) were increased, but the eNOS activity (Figure 6(c)) was decreased in aortic tissues dissected from diabetic mice, which were prevented by salusin-*β* knockdown. The aortic tissues from diabetic mice displayed lower protein level of phosphorylated eNOS, but higher iNOS, IL-1*β*, MCP-1, TNF-*α*, and VCAM-1 at both protein (Figure 6(d)) and mRNA (Figure 6(e)) levels, which were all reversed by gene silencing of salusin-*β*.

### 3.9. Salusin-*β* Knockdown Reduced ROS Production *In Vivo*

**Figure 7:**
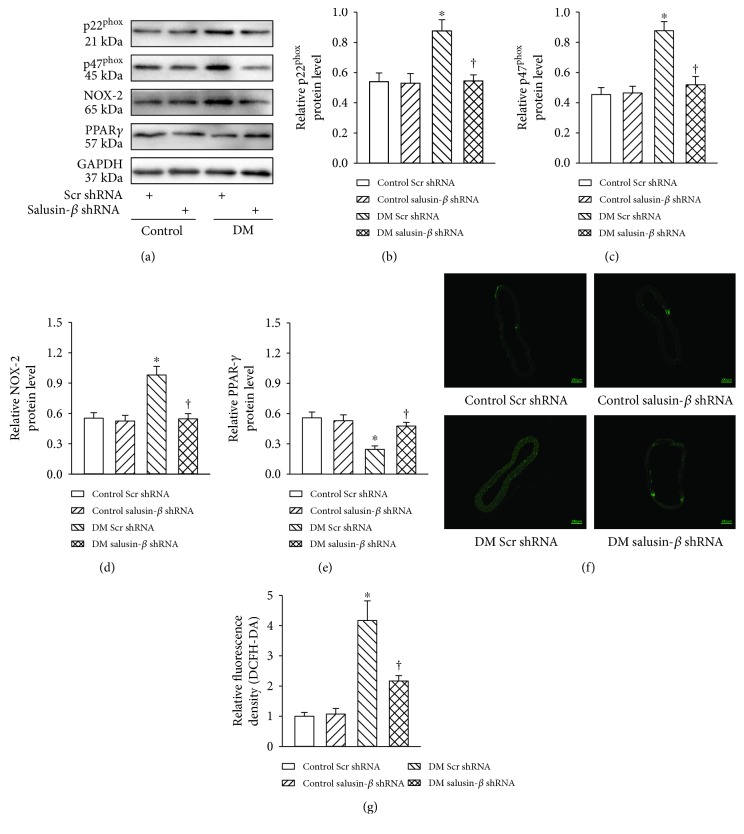
Salusin-*β* knockdown reduced ROS production *in vivo*. Intravenous injection of adenoviral vectors encoding salusin-*β* shRNA (Ad-salusin-shRNA, 2.0 × 10^10^ plaque-forming units) or scramble shRNA (Ad-Scr-shRNA) were carried out 8 weeks after STZ injection. The measurements were made 2 weeks after the first adenovirus transfer. (a) Blots showing the protein expressions of p22^phox^, p47^phox^, NOX-2, and PPAR*γ*. Bar group showing the protein levels of p22^phox^ (b), p47^phox^ (c), NOX-2 (d), and PPAR*γ* (e). (f, g) Representative images of in situ superoxide detection with DCFH-DA staining. Values are mean ± SE. ^∗^*P* < 0.05 versus control scramble (Scr) shRNA or control salusin-*β* shRNA, ^†^*P* < 0.05 versus DM scramble (Scr) shRNA. *n* = 7 for each group.

The aorta from diabetic mice showed obvious changes in NADPH oxidase subunits p22^phox^ (Figures 7(a) and 7(b)), p47^phox^ (Figures 7(a) and 7(c)), and NOX-2 (Figures 7(a) and 7(d)), which were reversed by salusin-*β* shRNA. The decreased PPAR*γ* protein expression from diabetic aorta was ameliorated by shRNA-mediated silencing of salusin-*β* (Figures 7(a) and 7(e)). DCFH-DA fluorescent staining further confirmed that increased ROS production in diabetic aorta was attenuated by salusin-*β* knockdown (Figures 7(f) and 7(g)).

## 4. Discussion

Endothelial dysfunction is a requisite step in the progression of cardiovascular implications in diabetes [4]. The present study demonstrated that HG/HF induced inflammation response, excessive cellular ROS, and NOx generations accompanied by overexpression of salusin-*β* in HUVECs. Silencing of salusin-*β* prevented HG/HF-mediated endothelial injury via PPAR*γ* upregulation in HUVECs. Furthermore, downregulation of salusin-*β* ameliorated endothelium-dependent vasorelaxation of aortic rings from T2DM mice. These results suggested that salusin-*β* knockdown exerted protective effects on endothelial dysfunction in response to DM.

Salusin-*β* is identified to be expressed in human, rat, and mouse tissues such as vasculature, central nervous system, and kidneys [40–42]. The circulating salusin-*β* level is increased in diabetic patients [25], and salusin-*β* upregulation was observed in myocardium of diabetic rats [26]. In this study, we found that the salusin-*β* level was increased in both HG/HF-treated HUVECs and aorta from diabetic mice. These results indicated that hyperglycaemia may serve as a stimulator for salusin-*β* expression, and salusin-*β* may play a role in endothelial function in DM.

Hyperglycaemia acts on vascular endothelial cells to produce ROS to interrupt NO bioactivity, endothelium-dependent relaxation, and endothelial dysfunction [43]. The excessive ROS generation plays a functional role in endothelial dysfunction of DM [44]. NADPH oxidases are the major sources of ROS in vascular cells [4]. The NADPH oxidase subunits including NOX-2, p22^phox^, and p47^phox^ are major sources of ROS in the vascular wall [32]. Salusin-*β* promoted vascular injury-induced intimal hyperplasia via NOX-2-derived ROS activation, followed by NF-*κ*B/MMP-9 accumulation in VSMCs [16]. Salusin-*β* stimulates ROS production to increase lipid accumulation, monocyte adhesion in VSMCs [17]. Central blockade of salusin-*β* abated increased ROS levels in the paraventricular nucleus of hypertensive rats [45]. We found the increased superoxide anions, NADPH oxidase subunits including NOX-2, p22^phox^, and p47^phox^ in both HG/HF-incubated HUVECs and aorta from diabetic mice, which were suppressed by shRNA-mediated salusin-*β* silencing. These results indicated that salusin-*β* silencing-induced reduction of ROS may play protective effects on endothelial function.

Hyperglycaemia-triggered ROS induce endothelial cell apoptosis, NO bioactivity reduction, and endothelium-dependent relaxation impairment [46]. The downregulation of eNOS phosphorylation was involved in endothelial dysfunction [47]. Mounting evidence discloses that oxidative/nitrative stresses are critically involved in endothelial injury in T2DM [48, 49]. It is increasing recognized that low NOx concentrations have protective activities, whereas high NOx levels may lead to endothelial dysfunction and tissue injury [6, 9]. The superoxide can interact with NOx to cause the inactivation of NO and production of a highly cytotoxic molecule peroxynitrite, which plays a crucial role in the pathogenesis of diabetic vascular disease [9, 11]. We showed that the NOx levels, nitrotyrosine content, iNOS expression were markedly increased, while eNOS phosphorylation and activity was significantly reduced in HG/HF-treated HUVECs and aortic tissues from diabetic mice, which were eliminated by salusin-*β* knockdown. Moreover, salusin-*β* silencing improved endothelium-dependent vasorelaxation in diabetic aortic segments accompanied by decreased NOx levels, nitrotyrosine content, and iNOS expression and increased eNOS phosphorylation and activity. These results implied that the upregulated iNOS is the primary source of enzymes causing NOx overproduction, silencing of salusin-*β* alleviated nitrative stress through inhibiting the expression of iNOS. The endothelial protective effects of salusin-*β* knockdown in diabetic mice were largely attributed to direct inhibition of nitrative stress.

Inflammation response in endothelial cells is also involved in the pathogenesis of vascular complications of diabetes [4, 49]. Treatment of HUVECs with salusin-*β* stimulates the expressions of VCAM-1, MCP-1, and IL-1*β* [50–52]. In the present study, we showed that incubation of HUVECs with HG/HF upregulated the protein and mRNA levels of IL-1*β*, MCP-1, TNF-*α*, and VCAM-1, which were normalized by intervention of salusin-*β*. Similar to results from cell experiments *in vitro*, the increased IL-1*β*, MCP-1, TNF-*α*, and VCAM-1 expressions in aorta form diabetic mice were also obviously attenuated by silencing of salusin-*β*. These results hinted that salusin-*β* exerted a proinflammatory effect on vascular endothelial cells, and anti-salusin-*β* therapy may afford a novel strategy for treatment of endothelial inflammation in DM.

Peroxisome proliferator-activated receptor gamma (PPAR*γ*), as a transcriptional regulator of energy balance, is expressed in endothelial cells, and PPAR*γ* has a protective role in endothelial cells beyond its metabolic effects [53]. PPAR*γ* agonists are expected to ameliorate endothelial dysfunction in diabetes [54]. It is recently unveiled that salusin-*β* negatively regulated PPAR*γ* expressions at protein, mRNA, and gene promoter levels in VSMCs [55]. Our results identified that silencing of salusin-*β* substantially rescued the downregulated PPAR*γ* levels in HUVECs response to HG/HG medium, and similar results were seen in the aorta from diabetic mice. It is particularly worth noting that PPAR*γ* inhibitor T0070907 abolished the protective effect of salusin-*β* knockdown on the inflammation and oxidative/nitrative stresses in HG/HF-treated HUVECs. Moreover, the decreased PPAR*γ* protein expression from diabetic aorta was ameliorated by shRNA-mediated silencing of salusin-*β*. These data suggested that salusin-*β*-induced inhibition of PPAR*γ* expression in endothelial cells may be responsible for DM-related endothelial injury. It is important to point out that numerous studies have demonstrated a close link between oxidative/nitrative stresses and inflammation in endothelial dysfunction [56, 57]. Our results showed that DM-induced oxidative/nitrative stresses and inflammation in endothelial cells were all ameliorated by knockdown of salusin-*β*. Due to the complexity and comprehensiveness of pathogenesis of DM, we speculated that these phenotypes regulated by salusin-*β* were potentially linked, and thus synergistically participated in endothelial dysfunction in T2DM. Activation of PPAR*γ* is well established to reduce inflammation and oxidative stress and improve endothelial function in diabetic conditions [58, 59]. Our results verified that PPAR*γ* was a pivotal effector molecule for salusin-*β*, and PPAR*γ* inhibitor T0070907 abolished the protective effects of salusin-*β* knockdown *in vitro*. We concluded that PPAR*γ*, as a downstream molecule of salusin-*β*, specifically modulated oxidative/nitrative stresses and inflammation in endothelial cells.

## 5. Conclusions

Taken together, silencing of salusin-*β* attenuated the HG/HF-induced endothelial cell dysfunction including inflammation and oxidative/nitrative stresses in HUVECs via regulation of PPAR*γ* (Figure S5). The destructive endothelium-dependent vasorelaxation of aortic rings from T2DM mice were obviously improved by knockdown of salusin-*β*. Our results provided the evidence that salusin-*β* was closely related with endothelial dysfunction in T2DM.

## Supplementary Material

FIGURE S1. Effects of high glucose/high-glucose/high-fat (HG/HF) on salusin-*β* expression in HUVECs. FIGURE S2. PPAR*γ* participated in the effects of salusin-*β* blockade on oxidative stress and inflammation in HG/HF-treated HUVECs. FIGURE S3. PPAR*γ* participated in the effects of salusin-*β* blockade on oxidative stress in HG/HF-treated HUVECs. FIGURE S4. Intravenous injection of adenoviral vectors encoding salusin-*β* shRNA had no significant on glucose tolerance test (GTT, a) and insulin tolerance test (ITT, b) in diabetic mice. FIGURE S5. A schematic overview about the effects of salusin-*β* and the involved mechanisms in DM-induced endothelial dysfunction. TABLE S1. Primer for RT-PCR analysis in HUVECs. TABLE S2. Primer for RT-PCR analysis in mice.
