# Supplementary figures and images for: Salusin-β Is Involved in Diabetes Mellitus-Induced Endothelial Dysfunction via Degradation of Peroxisome Proliferator-Activated Receptor Gamma
Source: Oxid Med Cell Longev. 2017 Nov 19;2017:6905217. doi: 10.1155/2017/6905217 (PMC5735326; doi:10.1155/2017/6905217)

## Slide 1
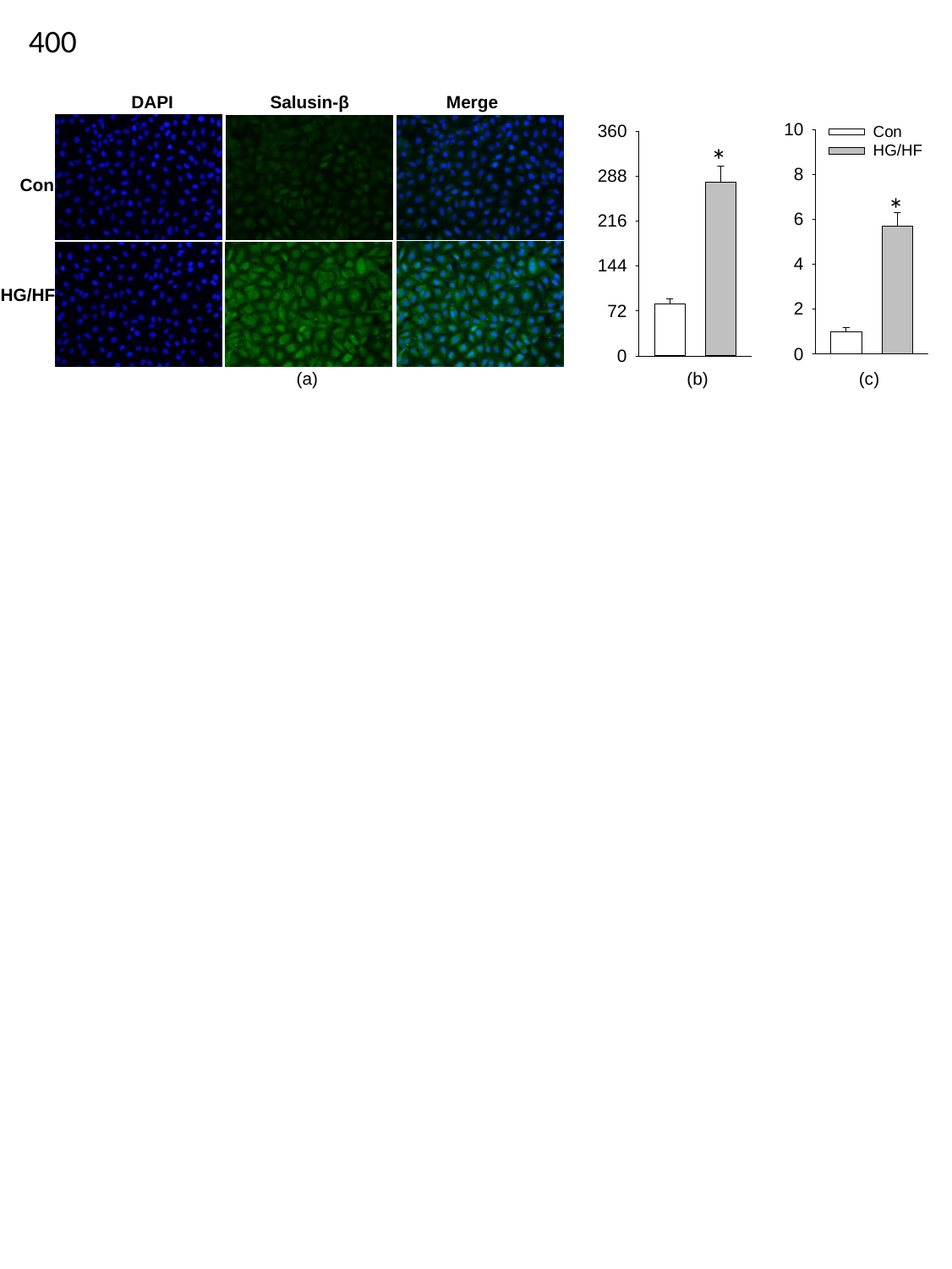

400
 DAPI Salusin-β Merge
*
Con
*
HG/HF
(a) (b) (c)

Supplement: Supplementary file 1 — FIGURE S1. Effects of high glucose/high-glucose/high-fat (HG/HF) on salusin-β expression in HUVECs. FIGURE S2. PPARγ participated in the effects of salusin-β blockade on oxidative stress and inflammation in HG/HF-treated HUVECs. FIGURE S3. PPARγ participated in the effects of salusin-β blockade on oxidative stress in HG/HF-treated HUVECs. FIGURE S4. Intravenous injection of adenoviral vectors encoding salusin-β shRNA had no significant on glucose tolerance test (GTT, a) and insulin tolerance test (ITT, b) in diabetic mice. FIGURE S5. A schematic overview about the effects of salusin-β and the involved mechanisms in DM-induced endothelial dysfunction. TABLE S1. Primer for RT-PCR analysis in HUVECs. TABLE S2. Primer for RT-PCR analysis in mice. [file 6905217.f1.pptx]

## Slide 1
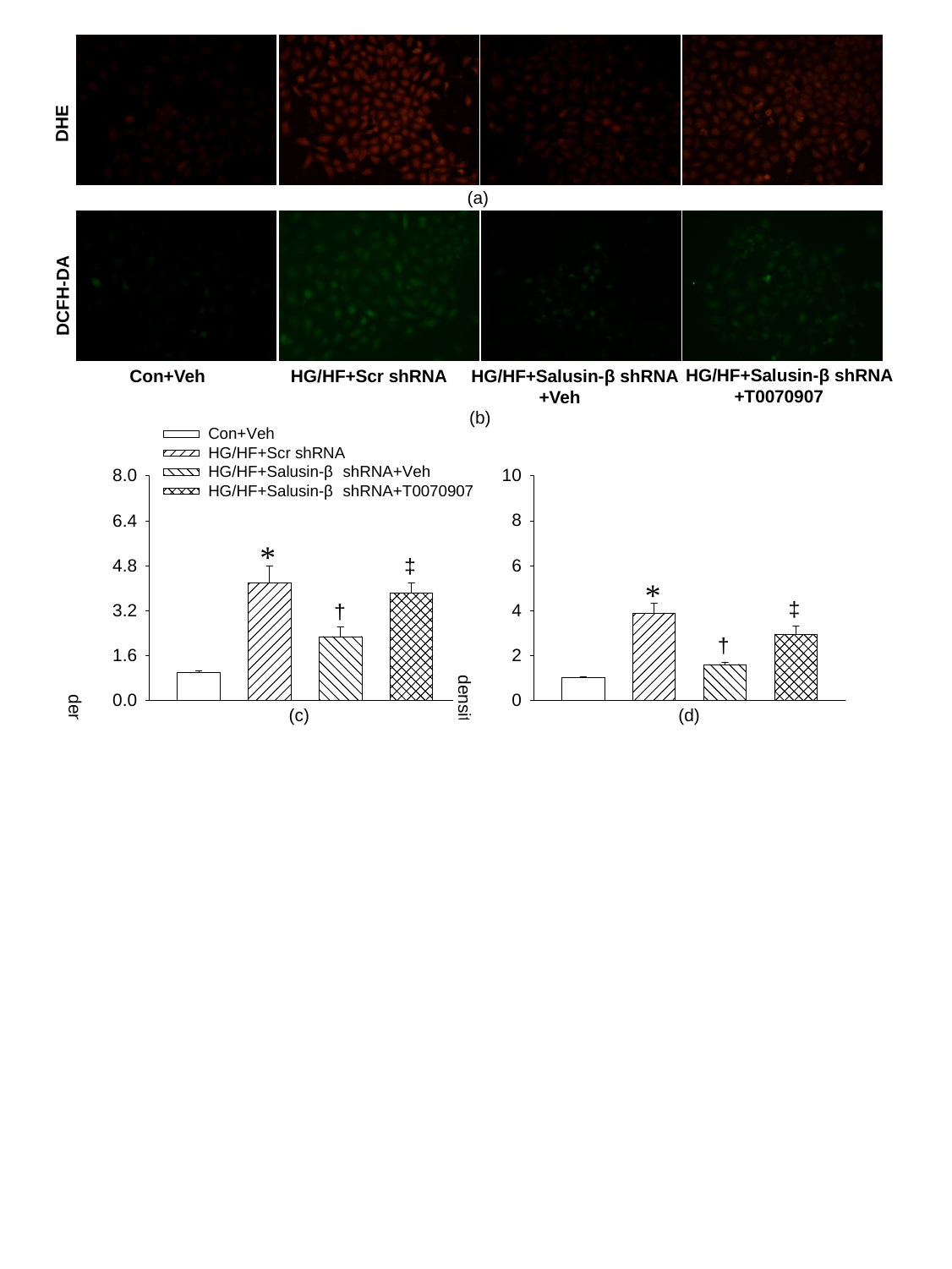

DHE
(a)
DCFH-DA
HG/HF+Salusin-β shRNA
 +T0070907
HG/HF+Salusin-β shRNA
 +Veh
Con+Veh
HG/HF+Scr shRNA
(b)
*
‡
*
‡
†
†
(c) (d)

Supplement: Supplementary file 3 [file 6905217.f3.pptx]

## Slide 1
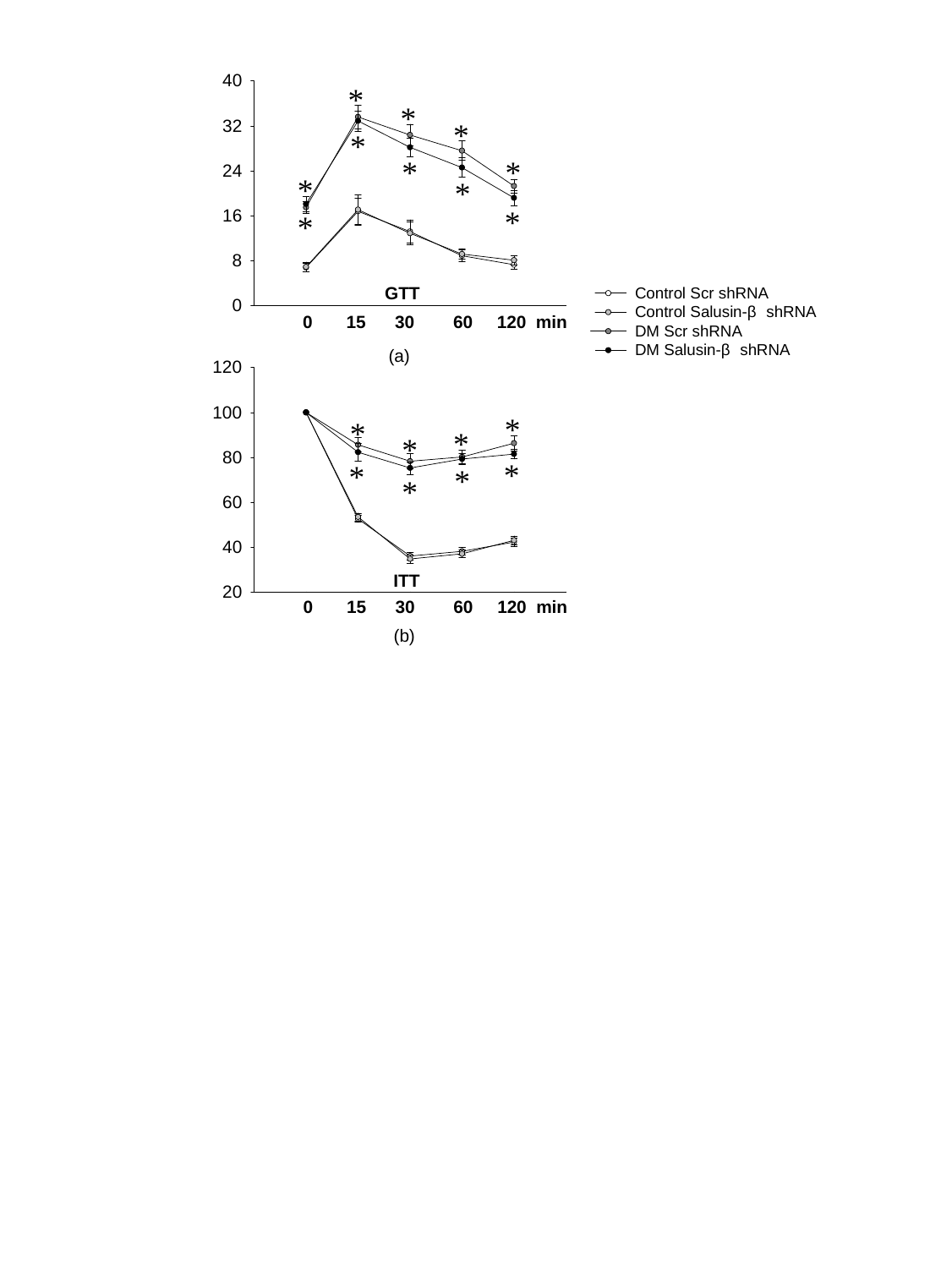

*
*
*
*
*
*
*
*
*
*
 GTT
0 15 30 60 120 min
(a)
*
*
*
*
*
*
*
*
 ITT
0 15 30 60 120 min
(b)

Supplement: Supplementary file 4 [file 6905217.f4.pptx]
