# Supplementary material for: Salusin-β Is Involved in Diabetes Mellitus-Induced Endothelial Dysfunction via Degradation of Peroxisome Proliferator-Activated Receptor Gamma
Source: Oxid Med Cell Longev. 2017 Nov 19;2017:6905217. doi: 10.1155/2017/6905217 (PMC5735326; doi:10.1155/2017/6905217)
Supplement: Supplementary file 2 [file 6905217.f2.pptx]

## Slide 1
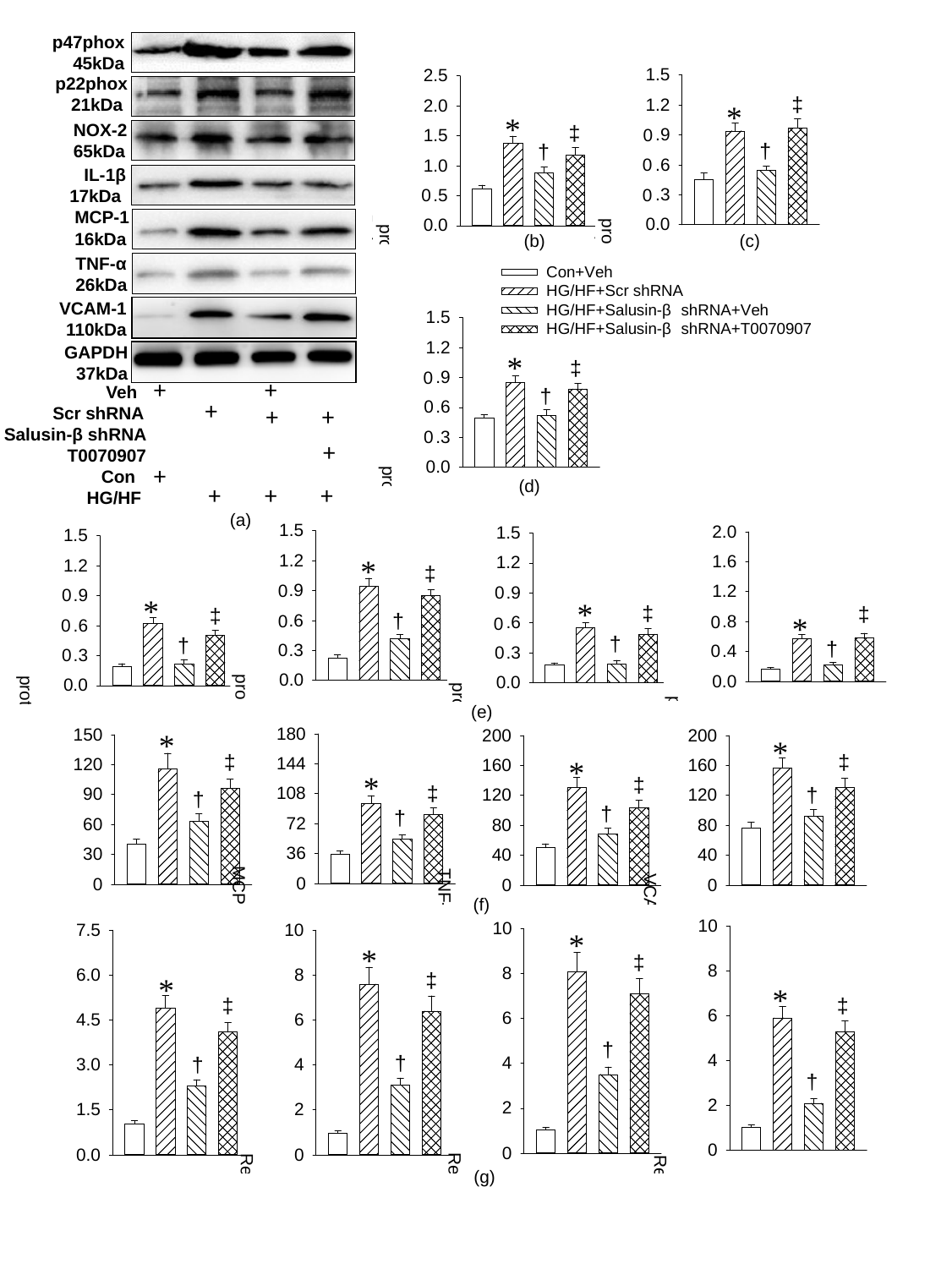

p47phox
45kDa
p22phox
21kDa
‡
*
*
NOX-2
65kDa
‡
0
†
†
0
IL-1β
17kDa
0
0
MCP-1
16kDa
(b) (c)
TNF-α
26kDa
VCAM-1
110kDa
GAPDH
37kDa
*
‡
0
 + +
 Veh
 Scr shRNA
Salusin-β shRNA
 T0070907
 Con
 HG/HF
†
 +
0
 + +
0
 +
 +
(d)
+ + +
(a)
*
‡
0
0
0
*
*
‡
‡
‡
†
*
0
0
0
0
†
†
†
0
0
0
0
(e)
*
*
‡
‡
*
*
‡
‡
†
†
†
†
(f)
*
*
‡
‡
*
*
‡
‡
†
†
†
†
(g)
