# Supplementary material for: Salusin-β Is Involved in Diabetes Mellitus-Induced Endothelial Dysfunction via Degradation of Peroxisome Proliferator-Activated Receptor Gamma
Source: Oxid Med Cell Longev. 2017 Nov 19;2017:6905217. doi: 10.1155/2017/6905217 (PMC5735326; doi:10.1155/2017/6905217)
Supplement: Supplementary file 5 [file 6905217.f5.pptx]

## Slide 1
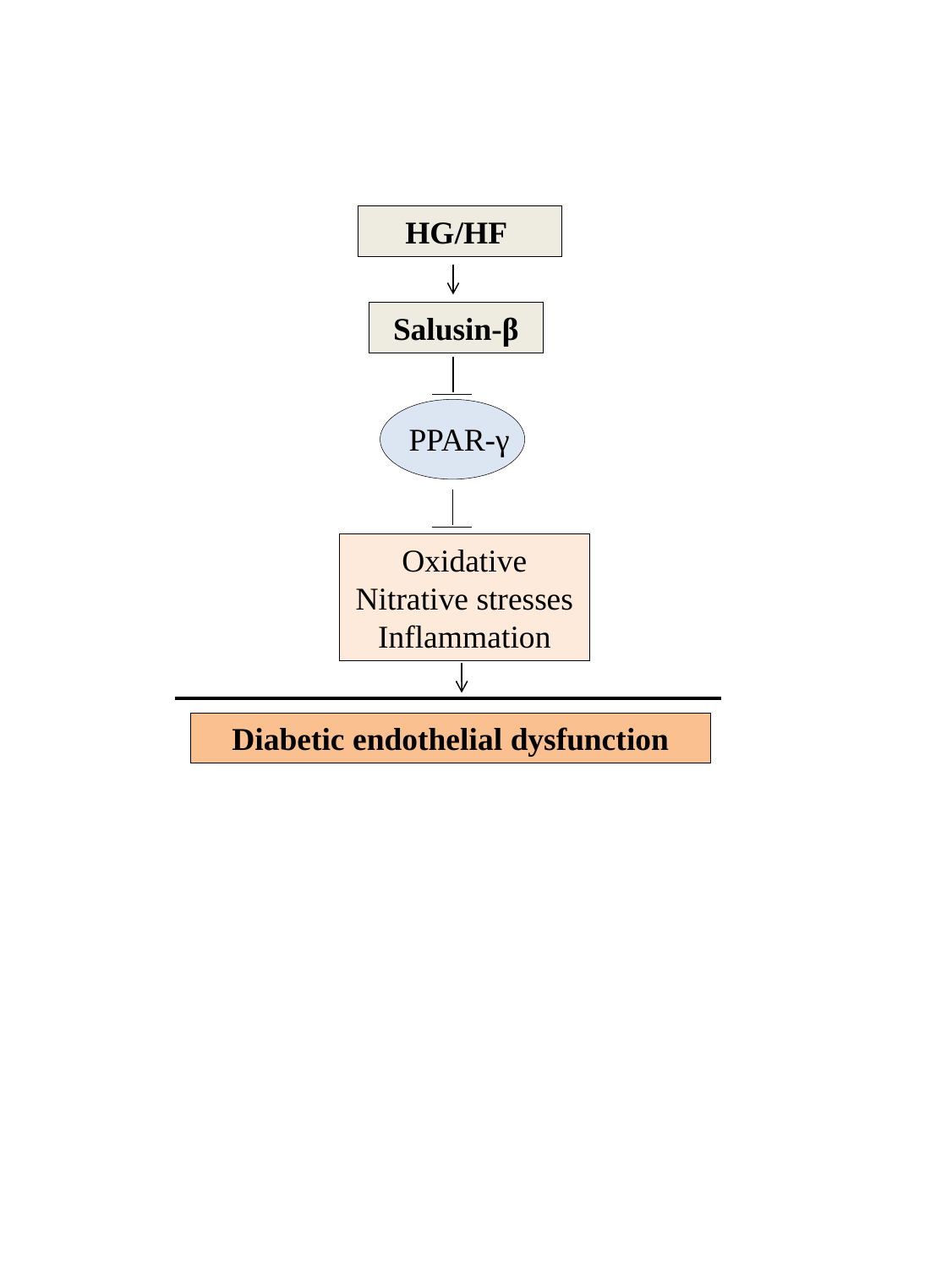

HG/HF
Salusin-β
PPAR-γ
Oxidative
Nitrative stresses
Inflammation
Diabetic endothelial dysfunction
