# Supplementary material for: Salusin-β Is Involved in Diabetes Mellitus-Induced Endothelial Dysfunction via Degradation of Peroxisome Proliferator-Activated Receptor Gamma
Source: Oxid Med Cell Longev. 2017 Nov 19;2017:6905217. doi: 10.1155/2017/6905217 (PMC5735326; doi:10.1155/2017/6905217)
Supplement: Supplementary file 6 [file 6905217.f6.docx]

**Supplementary Materials**

(5 supplementary figures and 2 supplementary tables)

**Salusin-β is involved in diabetes mellitus-induced endothelial dysfunction via degradation of peroxisome proliferator-activated receptor gamma**

Hai-Jian Sun^1,2^*, Dan Chen^1^*, Pei-Yao Wang^2^*, Ming-Yu Wan^2^*, Chen-Xing Zhang^2^, Zhi-Xuan Zhang^2^, Wei Lin^2^, Feng Zhang^1&^

1 Department of Physiology, Nanjing Medical University, Nanjing, Jiangsu 211166, P.R. China.

2 Department of Basic Medicine, Wuxi School of Medicine, Jiangnan University, Wuxi, Jiangsu 214122, P.R. China.

FIGURE S1. Effects of high glucose/high-glucose/high-fat (HG/HF) on salusin-β expression in HUVECs. HUVECs were cultured in control or HG/HF-containing medium for 24 h. (a) Immunofluorescence staining showing the protein expression of salusin-β (green) in aorta. Nuclei were stained by DAPI (blue). (b) The protein levels of salusin-β detected by enzyme linked immunosorbent assay (ELISA). (c) The mRNA levels of salusin-β detected by real-time PCR. Values are mean±SE. * P < 0.05 vs. Control (Con). n = 6 for each group.

FIGURE S2. PPARγ participated in the effects of salusin-β blockade on oxidative stress and inflammation in HG/HF-treated HUVECs. (a) Represented blots showing the protein expressions of p47^phox^, p22^phox^, NOX-2, IL-1β, MCP-1, TNF-α, and VCAM-1. Bar group showing the relative quantification of phosphorylated p22^phox^ (b), p47^phox^ (c) and NOX-2 (d). (e) Bar group showing the relative protein quantification of IL-1β, MCP-1, TNF-α, and VCAM-1. (f) Bar group showing the mRNA levels of IL-1β, MCP-1, TNF-α, and VCAM-1 determined with ELISA. (g) Bar group showing the mRNA levels of IL-1β, MCP-1, TNF-α, and VCAM-1 determined with real-time PCR. Values are mean±SE. * P < 0.05 vs. Control (Con) + Vehicle (Veh), † P < 0.05 vs. HG/HF+ Scramble (Scr) shRNA, ‡ P < 0.05 vs. HG/HF+salusin-β shRNA + Vehicle (Veh). n = 6 for each group.

FIGURE S3. PPARγ participated in the effects of salusin-β blockade on oxidative stress in HG/HF-treated HUVECs. (a,c) Represented images showing the levels of superoxide anions detected by DHE staining. (b,d) Represented images showing the ROS levels detected by DCFH-DA staining. Values are mean±SE. * P < 0.05 vs. Control (Con) + Vehicle (Veh), † P < 0.05 vs. HG/HF+ Scramble (Scr) shRNA, ‡ P < 0.05 vs. HG/HF+salusin-β shRNA + Vehicle (Veh). n = 6 for each group.

FIGURE S4. Intravenous injection of adenoviral vectors encoding salusin-β shRNA had no significant on glucose tolerance test (GTT, a) and insulin tolerance test (ITT, b) in diabetic mice. Intravenous injection of adenoviral vectors encoding salusin-β shRNA (Ad-Salusin-shRNA, 2.0 × 10^10^ plaque-forming units) or scramble shRNA (Ad-Scr-shRNA) were carried out 8 weeks after STZ injection. The measurements were made 2 weeks after the first adenovirus transfer. Values are mean±SE. * P < 0.05 vs. Control Scramble (Scr) shRNA or Control Salusin-β shRNA, n = 7 for each group.

FIGURE S5. A schematic overview about the effects of salusin-β and the involved mechanisms in DM-induced endothelial dysfunction.

TABLE S1 Primer for RT-PCR analysis in HUVECs

| Primers | Sequences (5’-3’) |
| --- | --- |
| GAPDH (Forward) | CCACATCGCTCAGACACCAT |
| GAPDH (Reverse) | CCAGGCGCCCAATACG |
| Salusin-β (Forward) | GGGTGGTATACGGGACCAAT |
| Salusin-β (Reverse) | ACAGCCTGGACAACCTCATC |
| TNF-α (Forward) | TGCTGCACTTTGGAGTGATCG |
| TNF-α (Reverse) | TGTCACTCGGGGTTCGAGAAG |
| IL-1β (Forward) | TCCAGGGACAGGATATGGAG |
| IL-1β (Reverse) | TCTTTCAACACGCAGGACAG |
| MCP-1 (Forward) | GATGCAATCAATGCCCCAGTC |
| MCP-1 (Reverse) | TCCTTGGCCACAATGGTCTTG |
| VCAM-1 (Forward) | TTGCTGACAGCTGACCTTTG |
| VCAM-1 (Reverse) | TTTAGGCCACATTGGGAAAG |

Note: GAPDH, glyceraldehyde phosphate dehydrogenase; TNF-α, tumor necrosis factor-α; IL-1β, interleukin-1β; MCP-1, monocyte chemoattractant protein 1; VCAM-1, vascular cellular adhesion molecule-1; PPAR-γ, peroxisome proliferator-activated receptor γ.

TABLE S2 Primer for RT-PCR analysis in mice

| Primers | Sequences (5’-3’) |
| --- | --- |
| GAPDH (Forward) | TCAACGGCACAGTCAAGG |
| GAPDH (Reverse) | ACCAGTGGATGCAGGGAT |
| Salusin-β (Forward) | CACTTCCCCCACCCCAGCCACA |
| Salusin-β (Reverse) | CCGACACTCCGTTCATCTCACT |
| TNF-α (Forward) | GTCCCCAAAGGGATGAGAAG |
| TNF-α (Reverse) | CACTTGGTGGTTTGCTACGA |
| IL-1β (Forward) | CCCAACTGGTACATCAGCACCTC |
| IL-1β (Reverse) | GACACGGATTCCATGGTGAAGTC |
| MCP-1 (Forward) | CCCCACTCACCTGCTGCTACT |
| MCP-1 (Reverse) | TTTACGGGTCAACTTCACATTCAA |
| VCAM-1 (Forward) | GCCCTCACTTGCAGCACTAC |
| VCAM-1 (Reverse) | TCCTCACCTTCGCGTTTAGT |

Note: GAPDH, glyceraldehyde phosphate dehydrogenase; TNF-α, tumor necrosis factor-α; IL-1β, interleukin-1β; MCP-1, monocyte chemoattractant protein 1; VCAM-1, vascular cellular adhesion molecule-1; PPARγ, peroxisome proliferator-activated receptor γ.
